# Intracerebral Hemorrhage, Oxidative Stress, and Antioxidant Therapy

**DOI:** 10.1155/2016/1203285

**Published:** 2016-04-14

**Authors:** Xiaochun Duan, Zunjia Wen, Haitao Shen, Meifen Shen, Gang Chen

**Affiliations:** ^1^Department of Neurosurgery, The First Affiliated Hospital of Soochow University, 188 Shizi Street, Suzhou 215006, China; ^2^Department of Neurosurgery, Yangzhou No. 1 People's Hospital, No. 45, Taizhou Road, Yangzhou 225001, China

## Abstract

Hemorrhagic stroke is a common and severe neurological disorder and is associated with high rates of mortality and morbidity, especially for intracerebral hemorrhage (ICH). Increasing evidence demonstrates that oxidative stress responses participate in the pathophysiological processes of secondary brain injury (SBI) following ICH. The mechanisms involved in interoperable systems include endoplasmic reticulum (ER) stress, neuronal apoptosis and necrosis, inflammation, and autophagy. In this review, we summarized some promising advances in the field of oxidative stress and ICH, including contained animal and human investigations. We also discussed the role of oxidative stress, systemic oxidative stress responses, and some research of potential therapeutic options aimed at reducing oxidative stress to protect the neuronal function after ICH, focusing on the challenges of translation between preclinical and clinical studies, and potential post-ICH antioxidative therapeutic approaches.

## 1. Introduction

Intracerebral hemorrhage (ICH) is a serious cerebrovascular condition leading to high mortality and morbidity in adults. The global incidence of ICH is increasing year by year, with a trend towards growing incidence at a younger age [1]. Despite significant progress in clinical treatment, the 5-year mortality rate remains over 50% (52% for males, 56% for females) in ICH patients older than 45 years [2]. Even after surgical treatment, 20% of ICH patients experience varying degrees of neurological dysfunction, requiring long-term hospitalization and rehabilitation [3]. Thus, ICH not only causes serious morbidity and mortality in patients, but also incurs a serious burden on families and society. The pathological mechanisms of hematoma after ICH within brain parenchyma triggers a series of adverse events causing SBI and severe neurological deficits [4]. In recent years, progress has been made in ICH research. In particular, it has been established that oxidative stress plays an important role in SBI after ICH, which leads to irreversible disruption of the components of the neurovascular unit, constituting gray and white matter, and is followed by blood brain barrier disruption and deadly brain edema with massive brain cell death [5]. Antioxidative treatment aimed at preventing or reducing oxidative stress has provided new insights into ICH therapy. In this paper, we review the mechanism of oxidative stress after ICH and the detection of biomarkers; we also summarize in detail the latest developments in antioxidative stress therapy.

## 2. Definition of Oxidative Stress

Oxidative stress describes a state when the body responds to various harmful stimuli and produces excessive amounts of reactive oxygen free radicals, known as reactive oxygen species (ROS), and reactive nitrogen radicals, known as reactive nitrogen species (RNS). Oxidative stress reflects an imbalance between the systemic manifestation of reactive oxygen species and a biological system's ability to readily detoxify the reactive intermediates or to repair the resulting damage. This leads to accumulation of ROS and RNS in the body or in cells, causing cell toxicity and eventually leading to tissue damage. The damage to intracellular proteins, lipids, and DNA caused by oxidative stress products such as reactive free radicals and peroxides is an important factor [6] in aging and is also involved in the pathogenesis of cancer [7], Parkinson's disease [8], Alzheimer's disease [9], atherosclerosis [10], heart failure [11], sickle cell disease [12], lichen planus [13], vitiligo [14], infection [15], chronic fatigue syndrome, and other diseases. Although excessive oxidative stress is considered harmful, reactive oxygen is beneficial for maintaining the normal physiological activity of the body and plays a role in defense, killing pathogens by regulating the immune system [16]. Short-term activation of oxidative stress may have an important role in preventing aging. For example, hydrogen peroxide, the most common form of active oxygen, promotes apoptosis at high concentrations, inducing antioxidant enzyme expression and increasing the antioxidant capacity of cells at low concentration [17]. The outcome of oxidative stress depends on the degree of damage in the balance between the oxidative and antioxidative responses of the body. Cells are capable of self-regulation of mild oxidative stress changes and restoring cell homeostasis. However, severe oxidative stress can lead to cell death, and it has been reported that although moderate oxidative stress can cause cell apoptosis, intense oxidative stress may lead to necrosis [18].

## 3. Oxidative Stress and ICH

**Figure 1:**
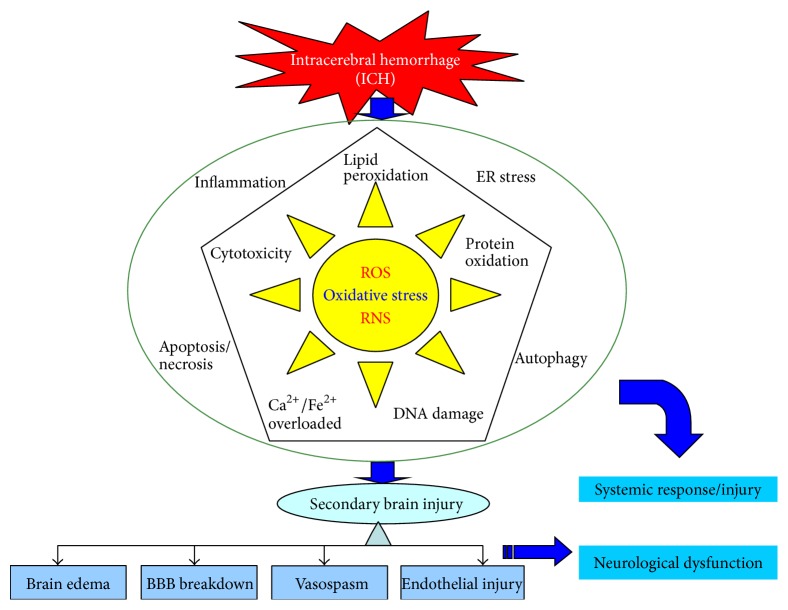
Schematic representation of major intracellular pathway in the role of reactive oxygen species radicals in hemorrhagic stroke. ROS: Reactive oxygen species; RNS: reactive nitrogen species; ICH: intracerebral hemorrhage; ER stress: endoplasmic reticulum stress; BBB: blood brain barrier.

Oxidative stress plays an important role in SBI after ICH [19]. Oxidative stress is involved not only in the pathological process of ICH, but also at various important stages of pathophysiological response during ICH [5]. A variety of pathways can induce the generation of free radicals after ICH, of which there are two major pathways. First, blood cell decomposition products such as iron ions, heme, and thrombin can induce the production of free radicals. Experimental results show that divalent iron ions can interact with lipid and generate free radicals, leading to nerve damage [20, 21]. Second, inflammatory cells, such as microglia and neutrophils, can generate free radicals. During the inflammatory response following ICH, neutrophils are stimulated and activated, resulting in outbreak of the respiratory chain, releasing large amounts of reactive oxygen species, nitric oxide, and so on, and the excessive consumption of superoxide dismutase (SOD) and the occurrence of lipid peroxidation [22]. Damage to nerve cells caused by free radicals manifests in a number of ways, with free radicals involved in pathological processes ranging from cell membrane damage to DNA interruption or even apoptosis [23]. Cell damage caused by oxygen free radicals is due to the induction of lipid peroxidation. The lipid-rich brain tissue is particularly sensitive to oxygen free radicals that can enhance lipid peroxidation, cause membrane damage, and increase cell membrane permeability and calcium ion influx [23]. In the meantime, cross-linking and polymerization of membrane lipids will occur due to lipid peroxidation, which will indirectly inhibit the activities of membrane proteins such as calcium pumps, sodium pumps, and Na^+^/Ca^2+^ exchangers [24]. This leads to a further increase in intracellular calcium concentration which subsequently stimulates mitochondrial calcium pumps to take in calcium. Calcium and phosphate in the mitochondria combine and form insoluble calcium phosphate, which causes interference in mitochondrial oxidative phosphorylation and leads to a decrease in ATP production [25]. Meanwhile, increased intracellular calcium ion concentration can activate phospholipase, promoting membrane phospholipid decomposition and causing damage to the structure of cell and organelle membranes [26, 27]. In summary, free radicals are the major killers of hemorrhagic brain tissue, with considerable recent research indicating that free radicals are closely related to brain injury and disorders caused by bleeding (Figure 1).

### 3.1. Oxidative Stress and Inflammation following ICH

Inflammation and oxidative stress are closely related. Oxidative stress induces inflammation, while inflammation causes damage through oxidative stress [28]. ROS can induce the expression of acute proinflammatory cytokines directly such as Tumor Necrosis Factor (TNF-*α*) and Interleukin-10 (IL-10) and also activate nuclear factor-*κ*B(NF-*κ*B) which plays the vital role in inflammation reaction [29, 30]. On the other hand, proinflammatory cytokines can induce the production of ROS [29]; thus, a positive feedback cycle is formed. In rats, hemoglobin mediates oxidative and nitration stress after ICH, with the nitration stress induce perihematomal edema which may be involved in neurovascular damage and neurological deficit [31]. Oxidative stress may initiate the upregulation of MMP-9 levels in brain damage after ICH [32]. MMP-9 levels in animal models have largely shown detrimental correlations with mortality, clinical outcome, hematoma volume, and SBI. Animal models and clinical studies have established a timeline for MMP-9 expression and corresponding perihematomal edema after ICH that include an initial peak on days 1–3 and a secondary peak on day 7. Another study demonstrated that MMP-9 expression was increased, accompanied by elevated TNF-*α* and IL-1*β* levels, and cerebral edema and SBI were aggravated [33]. Clinical studies suggest that MMP-9 may be detrimental in the acute phase through destruction of basal lamina, activation of vascular endothelial growth factor, and activation of apoptosis but assist in recovery in the subacute phase through angiogenesis. Therefore, MMP-9 activity may have dual role and temporal profile in post-ICH [34].

Additional studies have shown that prostaglandin-mediated inflammatory mechanisms are involved in secondary brain damage after ICH. In a collagenase-induced ICH model in mice, prostaglandin E2 receptor 1 (EP1R) was expressed in neurons and axons but not in astrocytes and microglia. EP1R agonists induce brain edema, cell death, neurodegeneration, neuroinflammation, and behavioral defects, while EP1R suppression protects the brain. Research has confirmed that the inhibition effect of EP1R is mainly through the reduction of Scr enzyme phosphorylation levels and MMP-9 activation, thus attenuating oxidative stress and white matter damage [35].

Peroxiredoxin I (PrxI) and heme oxygenase-1 (HO-1) are considered to be oxidative stress- and heme-related proteins, and heme inhibits PrxI antioxidant activity. Studies have shown that after ICH the expression of HO-1 and PrxI was induced around the hemorrhagic region. In the acute bleeding phase, PrxI and HO-1 are mainly expressed in microglia, while in subacute and chronic phases expression is mainly in astrocytes [36]. However, Prx1 is multifunctional protein important for cell protection against oxidative stress, but also works to facilitate production of prostaglandins E2 and D2 (PGE2 and PGD2) through nuclear factor- (erythroid-derived 2) like 2 (Nrf2) [37]. Prx family proteins released extracellularly from necrotic brain cells in the ischemic brain, which can induce the production of inflammatory cytokines through Toll-like receptors 2 and 4, prompt the release of high mobility group box 1 (HMGB1) protein and inhibit the activation of phagocytic cells and promoting cell death, even though intracellular Prxs have been shown to be neuroprotective. Extracellular Prxs are involved in brain ischemia-reperfusion injury by activating inflammatory pathways [38]. Acute inflammation is regulated by the time- and cell type-dependent production of cytokines and other signaling molecules including reactive oxygen species and prostaglandins [37]. In SBI after ICH, inflammatory damage, oxidative stress, calcium overload, iron overload, and cytotoxic injury form a complex cascade of reactions, among which inflammation and oxidative stress may play major roles; however, the relationship between inflammation and oxidative stress is complicated and needs further exploration.

### 3.2. Oxidative Stress and Endoplasmic Reticulum Stress after ICH

The pathological conditions may cause an imbalance between ER protein folding load and capacity after ICH, leading to the accumulation of unfolded proteins in the ER lumen, leading to a condition known as ER stress [5]. ER provides a unique oxidation-folding environment, favoring disulfide bond formation; thus, during the endoplasmic reticulum protein folding process, ROS will be produced [39]. Moderate activation of unfolded protein response after exposure to oxidation stress may be an adaption mechanism to protect cell function and survival, but ROS accumulation caused by excessive ER stress will further aggravate oxidative stress [39]. After ICH, both oxidative and ER stress levels are upregulated [5, 40], and alleviating either ER or oxidative stress will help improve secondary neuronal damage [41]. NADPH oxidase is thought to play an important contact role during the oxidative and ER stress process [42]. NMDA receptor is activated after ICH; a large amount of Ca^2+^ fluxes into the cells, leading to an NADPH oxidase and mitochondrial electron transport chain to produce superoxide [43, 44]. Therefore, NADPH oxidase generated by NMDA activation is considered a major superoxide source [45]. In addition, NADPH oxidase plays an important role in vascular brain disease. Using NADPH oxidase inhibitors and nonspecific ROS scavengers can reduce oxidative stress, improve cerebral vascular function, and reduce cerebral amyloid angiopathy-related microhemorrhages [46]. Neurons mainly express NOX2 in NADPH oxidase, which comprises gp91^phox^ catalytic subunit and p47^phox^ assembly subunit [43]. In a gp91^phox^ knockout mouse ICH model, brain damage and oxidative stress levels were significantly reduced [47]. Studies have shown that NOX-mediated oxidative stress is induced by unfolded protein response/ER stress, whereas ER stress-induced apoptosis can be blocked by knockout of NOX2 gene or antioxidant N- acetylcysteine [48, 49].

The PERK pathway is considered a molecule pathway which links oxidative and ER stress. Nrf2 induces considerable antioxidant gene expression [21]. After ICH, due to cytotoxicity mediated by heme, hemoglobin, and iron overload, Nrf2 is phosphorylated by PERK and then dissociates from the Nrf2/KEAP1 complex and enters into the nucleus to promote antioxidant gene expression, leading to a resistance to oxidative stress and playing a cell-protective role [21, 50]. Additionally, in Nrf2 knockout (Nrf2(−/−)) mice ICH model, injury volume was significantly larger in 24 h after induction of ICH, which correlated with neurological deficits. This exacerbation of brain injury was also associated with an increase in leukocyte infiltration, production of reactive oxygen species, DNA damage, and cytochrome c release during the critical early phase of the post-ICH period [51]. After subarachnoid hemorrhage (SAH), KEAP1-Nrf2-ARE pathway is activated, and after sulforaphane or tert-butylhydroquinone activates the Nrf2 pathway, NQO1 and GST-*α*1 levels are increased, thus playing a protective role in the brain [52–54]. In addition, Nrf2 and Transcriptional Factor 4 (ATF4) activate antioxidant response factor (ARE) by upregulating its expression [55, 56], indicating that the ER and oxidative stress signaling pathways have synergistic effects [57].

Endoplasmic reticulum oxidoreductase (ERO1*α*) forms a disulfide bond, promoting protein refolding and helping reduce ER stress. However, ERO1*α* activation transfers the electron to the oxygen molecule and produces ROS [58]. The endoplasmic reticulum stress marker CHOP, a downstream molecule of PERK, induces ERO1*α* expression and aggravates ER oxidation; on the contrary, in cells lacking CHOP, the ER stress level induced by ERO1*α* is reduced [59].

Other studies have shown that after ER stress inositol 1,4,5-trisphosphate receptors (IP3Rs) are activated and calcium release from the endoplasmic reticulum calcium storage is increased, leading to intracellular calcium overload and ROS production [60]. In addition, the elevated ROS level causes the activation of ryanodine receptor (RyRs), another endoplasmic reticulum Ca^2+^ release channel, and the release of Ca^2+^ from the ER [61, 62]. Thus, Ca^2+^ activates IP3Rs or RyRs as an input signal, aggravating intracellular calcium overload. After ICH, ER and oxidative stress activate ER Ca^2+^ release via RyRs and IP3Rs pathways, leading to neuronal toxicity and aggravating SBI [63].

### 3.3. Oxidative and Neural Cell Apoptosis or Necrosis after ICH

Apoptosis is a regulated cell death, which is also called programmed cell death (PCD). The main causes of nerve cell apoptosis are the release of thrombin during blood coagulation, the toxic effects of hematoma components and its degradation products, and the oxidative stress reaction in perihematoma [5]. ROS induce neuronal apoptosis through a variety of pathways. Excessive free radicals can cause the peroxidation of lipid, protein, and nucleic acid through direct and indirect pathways, leading to apoptosis [64]. Hypoxia, nitric oxide (NO), and ROS inducers can all cause the exposure of neuronal membrane phosphatidylserine [65]. Hydrogen peroxide and NO can lead to nuclear condensation and DNA fragmentation and have a synergistic effect on inducing neuronal apoptosis [66]. Additionally, NO can induce apoptosis of hippocampal and dopamine neurons [67, 68], and hydrogen peroxide can induce apoptosis through disrupting mitochondrial function and promoting proapoptosis gene expression [69, 70]. Oxidative stress induces apoptosis through pathways, such as the mitochondrial, death receptor, and endoplasmic reticulum stress pathways. It can also induce apoptosis by activating the mitogen-activated protein kinase pathway, activating NF-*κ*B and upregulating its expression, or activating caspases [71]. The intrinsic and extrinsic pathways of apoptosis are not necessarily independent of each other; some of the factors in both types of pathway may have a synergistic effect in the regulation of the apoptosis process, initiated by a single stimulator [72].

Hemorrhagic stroke proteins were shown to be involved in necrosis via proteomics approach [73]. Necrosis was formerly considered to be an accidental, unregulated form of cell death resulting from excessive stress, although it has been suggested that this is an oversimplistic view as necrosis may under certain circumstances involve the mobilization of specific transduction mechanisms [74]. Research suggests that superoxide generated by NADPH oxidase, besides that generated by the mitochondria, may contribute to the remarkable increase in the intracellular level of superoxide in the cells treated with menadione for 6 h resulting in the switch from apoptosis to necrosis [75]. Additionally, necrosis is characterized by plasma membrane rupture as well as nuclear and cellular swelling, other than regulated cell death. However, these findings suggest that DNA damage cytosolic reactive oxygen species (cROS) generation, and mitochondrial hyperactivation induced necrosis through a PARP1-dependent pathway, while generation of nitric oxide (NO) and mitochondrial ROS (mROS) remained unaffected [76]. Nevertheless, the relationship between necrosis and oxidative stress after ICH is still not fully clear.

Necroptosis was recently discovered as one form of programmed cell death (PCD) that shares characteristics with both necrosis and apoptosis. Necroptosis involves Fas/TNF-*α* death domain receptor activation and inhibition of receptor-interacting protein I kinase [77]. Recent study identified a novel role for the necroptosis inhibitor, necrostatin-1, in limiting neurovascular injury in tissue culture models of hemorrhagic injury [78]. Another study demonstrated that the specific inhibitor necrostatin-1 suppressed apoptosis and autophagy to exert these neuroprotective effects after ICH and that there existed a cross talk among necroptosis, apoptosis, and autophagy after ICH [79]. Moreover, necrostatin-1 reduced RIP1-RIP3 interaction and further inhibited microglia activation and TNF-*α* and IL-1*β* expression after ICH. These findings indicate that RIP1/RIP3-mediated necroptosis is an important mechanism of cell death after ICH [80]. In another study, hemin concentration dependently induced necroptotic cell death in cortical astrocytes within 5 h of treatment. Superoxide production paralleled the increase in iNOS expression, and inhibition of either iNOS (aminoguanidine or iminopiperdine) or superoxide (apocynin) significantly reduced cell death. Hemin-induced peroxidative injury was associated with a rapid depletion of intracellular glutathione (GSH), culminating in lipid peroxidation and cell death [81]. Together, these studies suggest a novel role for oxidative stress in necroptotic brain injury after ICH.

### 3.4. Oxidative Stress and Autophagy after ICH

Autophagy is a lysosomal degradation pathway that is essential for survival, development, and homeostasis, which plays a key role in diverse pathologies [82]. Recent studies indicate that autophagy is also involved in the pathological process of cerebral hemorrhage as a degradation process of proteins and organelles within the cells [83–87]. During this process, oxidative stress may contribute to autophagy formation. In addition, autophagy may reduce oxidative damage by engulfing or degrading stress products [88]. The intracellular mechanism which regulates autophagy via ROS levels can be summarized as transcriptional and posttranscriptional regulation, including various intracellular signaling pathways such as ROS-FOXO3-LC3/BNIP3 autophagy, ROS-Nrf2-P62 autophagy [82], ROS-HIF1-BNIP3/NIX autophagy, and ROS-TIGAR autophagy. Autophagy can also regulate ROS levels through a chaperone-mediated autophagy pathway, the mitochondrial autophagy pathway, and P62-mediated signaling pathways [89]. Autophagy plays a dual role in ischemic stroke pathological processes [90]. Our study also found that autophagy may play different roles in pathogenesis at different stages of cerebral hemorrhage, and further study of the relationship between oxidative stress and autophagy after ICH may provide a theoretical basis for elucidating the pathogenesis of cerebral hemorrhage [91].

## 4. Detection of the Level of Oxidative Stress

Biomolecules modified by ROS or any biological processes affected by ROS can be biomarkers of oxidative stress. Proteomics has provided a new approach for further study of biomarkers and mechanisms of the pathological process following cerebral hemorrhage [73]. A proteomic analysis of a collagenase-induced rat ICH model 3 hours after bleeding showed that, compared with a control group, there were 86 proteins expressed differently that were mainly proteins involved in autophagy, ischemia, necrosis, apoptosis, calcium-activation, oxidative stress, cytokine secretion, and so on. Following ICH, superoxide dismutase-2 (SOD2), guanine nucleotide-binding protein, peroxiredoxin-1 (PRDX-1), and lactate dehydrogenase are downregulated, whereas peroxide catalase expression is upregulated [73]; additionally, PRDX-1 protein levels around the hematoma began to rise one day after ICH [36]. Even so, PRDX family proteins interact with intracellular antioxidant enzymes and play an important role in the cellular mechanism of SBI. Thus, it is suggested that expression of the oxidative stress biomarker following cerebral hemorrhage is a dynamic process, related to the volume of the cerebral hemorrhage hematoma, bleeding type, and different stages of bleeding. A considerable number of animal experiments have confirmed that after cerebral hemorrhage many biomarkers are expressed specifically in brain tissue and this has played an important role in deeper study of the disease [92, 93]. Ideally, a biomarker for a disease is detected in the target tissue and organs. However, the direct detection of various biological markers in the human brain is not realistic; therefore, for an oxidative stress biomarker to have clinical value it must not only be convenient for specimen collection but also accurately reflect the main source of ROS.

### 4.1. Detection of Lipid Peroxidation Markers

Lipid peroxide is an important product of brain damage and mainly derives from the secondary products of unsaturated fatty acid peroxidation of membrane phospholipid, causing structural and functional damage to the cell membrane. Currently, the presence of lipid peroxide in the peripheral blood is the most commonly used marker reflecting oxidative stress. The lipid peroxides malondialdehyde (MDA) and thiobarbituric acid reactive substance (TBARS) have been studied in most depth. Clinical studies and animal experiments have all shown that serum MDA levels increase rapidly at the early stage of ICH, and the level is closely related to clinical symptom severity [92, 94]. However, estimating lipid peroxide by measuring TBARS and MDA levels is not very accurate. The spectrophotometric method calculates the concentration based on the strength of chromogen produced from TBA reaction, but there are a number of substances in body fluids that can react with TBA, and MDA cannot specifically reflect oxidative stress levels after ICH [95]. Moreover, MDA and TBARS are derived from endogenous epoxide degradation rather than from the products of peroxidation; thus, the measured level of MDA and TBARS can easily lead to an overestimate of the free radical level.

Oxidized low-density lipoprotein (oxLDL) and lectin-like oxidized LDL receptor-1 (LOX-1) can damage endothelial function, enhancing platelet aggregation and promoting thrombosis, thus playing an important role in the pathogenesis of cerebral vasospasm after SAH [96, 97]. Although plasma oxLDL level is considered reflective of the oxidation state of the body [98], it is uncertain whether it can be used following ICH as a peripheral marker directly related to oxidative stress injury.

F2-isoprostane (F2IP), a lipid peroxidation marker which can be detected in both the blood and urine [99], is more stable and has higher sensitivity and specificity, when compared with MDA and TBARS. F2IP is usually measured by gas chromatography/mass spectrometry or high-performance liquid chromatography/mass spectrometry. Studies have shown that in SAH patients high levels of F2IP in cerebrospinal fluid are significantly correlated with poor prognosis.

Measuring F4-neuroprostanes (F4-NPs) can better assess the oxidative stress level of brain tissue after SAH and predict the prognosis of patients with SAH [100]. 8-iso-Prostaglandin F2*α* (8-iso-PGF2*α*) is an isomer derivative of F2-isoprostanes and is a reliable biomarker, currently used to evaluate oxidative stress and lipid peroxidation. It is present in blood, urine, and the fluid secretions of various tissues; it can also exist in tissue cells, either in its free form or esterified in phospholipids or other lipids, and it maintains a stable level in the body. Detecting 8-iso-PGF2*α* concentration in the blood of ICH patients has shown that 8-iso-PGF2*α* levels increased after ICH and are positively correlated with NIHSS (National Institute of Health Stroke Scale) score and hematoma volume. Analysis indicates that plasma level of 8-iso-PGF2*α* is an independent prognostic factor for ICH [101]. However, even though detection of plasma lipid hydroperoxides (ROOH) in patients with ICH was positively correlated with mortality within one week after ICH, regression analysis has shown that it cannot be used as a prognostic predictor for clinical outcome [102]. Plasma lipid peroxides are mostly induced by free radicals, and current detection techniques are subject to the limitations of serum or specimen handling and storage conditions, with a risk of self-oxidation of the specimens, leading to an abnormal increase in the detection levels of related biological markers. Thus, their value in clinical use is still to be determined.

### 4.2. Detection of DNA Oxidative Damage Markers

Mechanisms of DNA oxidative damage include oxidative modification and DNA cleavage: more specifically, these include DNA damage, especially 8-hydroxy-2-deoxyguanosine (8-OHdG), DNA breakage, and DNA-protein cross-linking.

8-OHdG is considered a reliable indicator for detecting the degree of oxidative stress, due to its relatively high content in DNA oxidation products and its high specificity [103, 104]. Research showed 8-OHdG turned positive 3 days after ICH, but the apurinic/apyrimidinic site began to increase 24 hours after hemorrhage, peaking in the first 3 days and beginning recovery from the seventh day [105]. Ku-70 and ku-86 proteins are DNA repair proteins that begin to decline 24 hours after ICH, reducing significantly 3 days after hemorrhage and returning to normal by the seventh day. Ku-70 and ku-86 proteins may participate in DNA damage after brain hemorrhage along with oxidative damage. Other studies focusing on the relationship between oxidative stress level detection and ICH prognosis have indicated that 8-OHdG level is closely related to prognosis 30 days after ICH [106].

### 4.3. Detection of Antioxidant Levels

Antioxidants are a group of compounds that can effectively resist or repair cellular oxidative damage to neuron lipids, DNA, and proteins. Many studies have used* in vivo* antioxidant levels as indirect markers for the evaluation of oxidative stress in stroke patients. Studies have shown that in ICH patients the activity of serum SOD, myeloperoxidase, and glutathione peroxidase (GSH-Px), glutathione S-transferase *α*1 (GST-*α*1), and quinone oxidoreductase 1 (NQO1) increases significantly after ICH or SAH, and this has associated with early brain damage and multiple organ damage [52, 95, 107]. In MRI-confirmed cerebral small vessel disease, the detection of blood inflammation and oxidative stress markers has shown that tumor necrosis factor receptor-*α* (TNFR*α*) and peroxidase are highly expressed in microbleeding lesions, while the expression of peroxidase is decreased in asymptomatic cerebral ischemia patients [108]. Antioxidant enzymes can help scavenge free radicals and reduce or eliminate oxidative damage; thus, determining the amount and activity of antioxidant enzymes in serum may reflect antioxidant activity in the body.

Nonenzymatic antioxidants are an important class of antioxidants that play important roles in the pathogenesis of cerebral hemorrhage. These include glutathione [53, 109], antioxidant vitamins [106, 110], uric acid [111], ubiquinone-reducing substances [54, 112], metallothionein [113], and thioredoxin system [114, 115]. However, methods for the detection of oxidative stress related nonenzymatic antioxidants in the peripheral blood are few; one study on the level of glutathione (GSH) in umbilical cord blood after neonatal periventricular hemorrhage found that cord blood GSH level was not related to intraventricular hemorrhage in low-birthweight neonates [116]. Uric acid is an antioxidant molecule of the body, but studies have found that although serum uric acid levels are related to ischemic stroke, they have no significant correlation with ICH [111]. Although both antioxidant enzymes and nonenzymatic antioxidant levels show changes in the cerebral hemorrhage patient, it still not possible to determine whether these changes are the cause or the result of oxidative stress after ICH, due to a lack of data on the antioxidant levels of patients before onset.

### 4.4. Detection of Other Oxidative Stress Markers

Free-iron-mediated oxidative stress reaction after ICH plays an important role in SBI [93, 117, 118], and measurement of free iron is an effective method for detecting oxidative stress. A novel multichannel spectral analysis method has been recently developed, to determine the concentration of iron and iron-containing protein polymer in and around hematoma. Results have shown a significant increase in total iron in hematoma, whereas in perihematoma increases are insignificant. Iron protein polymers in and around the hematoma were also significantly increased compared with a control group [119]. Iron plays an important role in generating free radicals following cerebral hemorrhage [118], aggravating brain damage by producing OH^−^ through the Fenton reaction. Transferrin is an iron binding protein which can bind to harmful free iron ions produced after ICH and transport them back to the cells. High-serum ferritin levels increase 3-4 days after ICH, which are independently associated with poor outcome in patients with ICH [120]. The study may suggest a neurotoxic effect of increased body iron stores in patients with hemorrhagic stroke [121], while the cerebrospinal fluid ferritin level continues to rise 5 days after ICH or SAH. Currently, cerebrospinal fluid ferritin level is considered a more reliable evaluating marker for hemorrhagic stroke [122]. Unfortunately, those results only proved in the experiment research that this is difficult to prove with biochemical assays that fail to differentiate between alterations that occur within the hematoma and perihematoma zone in human.

Bilirubin is a metabolic product of heme and the level of bilirubin in the body may reflect oxidative stress intensity after ICH [123, 124]. Determination of cerebrospinal fluid bilirubin and oxyhaemoglobin is important for the identification of spontaneous subarachnoid hemorrhage in CT-scan-negative patients [125].

3-Nitrotyrosine (3-NT) is a maker of ONOO^−^ formation. Under pathological conditions, the formation of 3-NT increases along with increase of reactive oxygen and nitrogen. Overexpressed NOS was concomitant with large quantities of 3-NT formation in the perihematomal after ICH as evaluated by Western blot and immunofluorescence. Moreover, levels of 3-NT in serum, which had a similar upregulation to that of in brain tissues, had a marked correlation with brain edema content and neurological deficits. Thus, 3-NT reflects the severity of SBI and predicting prognosis [126].

Oxidative stress is the result of imbalance between* in vivo* ROS generation and antioxidant defense, and measurement of a single oxidative stress product of antioxidant cannot fully reflect the oxidative stress level of the body; thus, measurement of plasma total antioxidant capacity (TAC) and total oxidant status (TOS) is more effective than other single measurement methods. Studies have found that TOS and NO serum levels in patients are significantly increased after cerebral hemorrhage, while TAC levels and catalase activity are significantly reduced. Therefore, the oxidative stress index (OSI) is increased [115, 127].

In summary, the most commonly used oxidative stress markers are lipid peroxides, peroxidation products of DNA and protein, and antioxidant substances in animal model. In the past few decades, many studies have been dedicated to finding biomarkers that can effectively reflect the level of oxidative stress after ICH in human (Table 1). However, results remain unsatisfactory due to the complexity of oxidative stress and the repeatability of antioxidants in a variety of reactions. This makes it difficult to find a biological marker with high sensitivity and specificity. In addition, due to limitations in sample collection, storage, and pretreatment method, the reproducibility of the measurement of ICH-related oxidative stress markers is poor. Consequently, improvement in methodology has become the prerequisite for a simple, accurate, and reliable biological marker. For now, a comprehensive analysis of various oxidative stress biological markers after ICH may better reflect the level of oxidative stress of the body.

## 5. Antioxidant Therapy after ICH

Maintaining redox equilibrium of the body is important for health maintenance and disease intervention, and the increase in oxidative stress levels and free radicals is related to antioxidation ability. Lowering the body level of ROS and RNS and increasing antioxidant capacity are two antioxidant treatment strategies after cerebral hemorrhage. Oxidative stress therapy after ICH mainly involves the use of natural and synthetic antioxidants (Table 2). Natural antioxidants include enzymes and nonenzyme antioxidants. Antioxidant enzymes include SOD, catalase (CAT), peroxidase, glutathione peroxidase (GSH-Px), and NADPH, and enhancing the activities of these can result in antioxidant effects. Nonenzymatic antioxidants are mostly derived from natural plants or their extracts and include vitamin C, vitamin E, glutathione, melatonin, carotenoids, resveratrol, ursolic acid, and microminerals such as copper, zinc, and selenium.

### 5.1. Recent Updates in the Treatment of ICH Using Natural Compounds

Nutrition and oxidative stress have a close, two-way relationship. On one hand, nutrients can produce reactive oxygen intermediates and free radicals during the metabolic process of the body, and transition metal trace elements such as iron and copper ions can promote ROS generation. On the other hand, a balanced diet and proper nutrition can enhance the antioxidant defense capability of the body, as some nutrients and food components have direct or indirect antioxidant effects [128]. Pyrroloquinoline quinone (PQQ) is a newly discovered water-soluble vitamin present as an antioxidant in food. Studies have shown that pretreatment of ICH rats with 10 mg/kg PQQ can effectively reduce neurological deficit, hematoma volume, and cerebral edema expansion. PQQ treatment reduces ROS production, increases the Bcl-2/Bax protein levels, and decreases the expression of apoptotic-factor-activated caspase-3. Therefore, in the treatment of cerebral hemorrhage, PQQ exerts its neuroprotective effect via antioxidative stress [129]. Melatonin is a hormone secreted by the pineal body. By scavenging free radicals, promoting antioxidation, and inhibiting lipid peroxidation, melatonin protects cell structure, prevents DNA damage, and reduces the level of peroxides in the body. We found that treating SAH rats with intraperitoneal injection of melatonin can regulate oxidative stress levels through the Nrf2-ARE signaling pathway and reduce early brain injury after SAH [112]. Plasma *α*-lipoic acid levels reflect the body's antioxidant levels, and treating SAH rats with *α*-lipoic acid raised the antioxidation level of the body, producing a neuroprotective effect [130]. Sulforaphane exerts a brain protection effect by inhibiting oxidative stress [131], the mechanism of which, activation of Nrf2 pathway, has been confirmed in ICH research [21]. Ursolic acid is present in triterpenoids in natural plants and can effectively reduce oxidative stress levels, increase antioxidative stress levels, and reduce early brain injury after SAH [132]. Sesamin, a lignan of sesame seed oil, is a promising natural product as a novel therapeutic strategy based on the regulation of microglial activities via inhibition of inducible NO synthase (iNOS) protein expression and accompanied by the activated p44/42 MAPK pathway in ICH [133]. Other natural plant extracts can be used as natural antioxidants, including resveratrol [134], astaxanthin [135], baicalein [136], astragaloside [137], proanthocyanidin [138], (−)-epigallocatechin-3-gallate [139], and apple polyphenol [97]. Studies in ICH animal models have shown that treatment using these extracts can exhibit neuroprotective effects by inhibiting oxidative stress, upregulating antioxidant levels, and reducing SBI after ICH.

### 5.2. Treatment of ICH Using Hydrogen and Active Hydrogen Compounds

Hydrogen sulfide exhibits a protective role in the brain, reducing oxidative stress after SAH [156]. Studies have found that hydrogen sulfide reduces ROS and MDA levels after SAH, reverses the decrease of SOD and GSH-Px, increases the expression level of hydrogen sulfide products CBS and 3MST in the brain tissues, and inhibits neuronal cell apoptosis by inhibiting the caspase-dependent apoptosis pathway. Hydrogen sulfide can also inhibit the secretion of inflammatory cytokines IL-1*β*, IL-10, and TNF-*α* after SAH. Recent antioxidation research on hydrogen shows that in an animal model of cerebral hemorrhage hydrogen inhalation or injecting hydrogen-rich saline can significantly reduce early brain damage and cerebral vasospasm following SAH [157, 158], and hydrogen inhalation preserved blood brain barrier disruption by prevention of mast cell activation after ICH [149]. Research has confirmed that hydrogen decreases the release of inflammatory cytokines by reducing lipid peroxides, increasing antioxidant enzyme activity, and reducing oxidative stress levels after SAH; meanwhile, inhalation of hydrogen reduces damage to the blood brain barrier following ICH and improves SBI [149, 150]. Recent clinical trials using hydrogen to treat acute cerebral anemia have proved that hydrogen treatment is safe [159]. Moreover, a random double-blind trial using hydrogen to treat SAH patients is underway, although not yet completed [160].

### 5.3. Targeted Therapy against Oxidative Stress Signaling Pathways

Rosiglitazone is a highly selective and potent agonist to peroxisome proliferator-activated receptor *γ*. Continuous treatment with 6 mg/kg rosiglitazone for 6 days reduces glutamate level, upregulates GLT-1 expression, reduces MDA and catalase levels, and ameliorates vasospasm following SAH, exhibiting a protective role in the brain [161]. Treatment with rosiglitazone in cerebral hemorrhage regulates antioxidation and anti-inflammatory effects, possibly through the interaction of Nrf-2, retinoid X receptor (RXR), and NF-*κ*B and reduces SBI [162]. Recently study shows RSG infusion therapy following minimally invasive surgery for ICH evacuation on perihematomal secondary brain damage, which might be more efficacious for reducing the levels of MMP-9 and secondary brain damage than minimally invasive surgery therapy alone [151].

Recent studies show that nitrate peroxide decomposition catalyst 5,10,15,20-tetrakis(4-sulfonatophenyl)porphyrinato iron (FeTPPS) reduced neurovascular damage and improved neurological deficits in a rat ICH model induced by caudate nucleus injection of hemoglobin; experimental data indicated that the activation of MMP-9 may be involved [31]. Moreover, effects of minocycline, a nonspecific MMP inhibitor, and pyrrolidine dithiocarbamate, an upstream regulator of MMPs, on MMP-9 activity and thereby the degree of ICH were also tested in another study; the result suggests that suppression of MMP-9 by minocycline [152] or pyrrolidine dithiocarbamate attenuated ICH, suggesting the therapeutic potential of MMP inhibitors in ICH [153]. However, attempts at MMP inhibition in spontaneous ICH have solely been made under experimental conditions and were associated with a wide range of possible side effects. Therefore, further comprehensive, elucidating investigations in this field are vital before any conclusions could be translated to humans [163].

Antioxidant research on statin drugs confirmed that statin treatment reduced plasma OxLDL levels in acute ischemic stroke patients [164]. In ICH rat model, atorvastatin showed significant effects in reducing the brain water content, blocking neuron apoptosis, and decreasing plasma MMP-9 levels [154]. Other studies have shown that atorvastatin induced a dose-dependent reduction of TNF-*α* and increase of IL-10 levels [155]. Thus that can decrease the brain injury and protect neurons in rats with ICH. However, recent literature suggests that statin treatment may not be effective in ICH [165].

As an iron-chelating agent, deferoxamine (DFX) binds to iron ions competitively, thereby preventing oxidation-reduction reaction of iron ions, reducing the generation of free radicals following hemorrhagic stroke and exhibiting a neuroprotective effect [166, 167]. Studies show that after ICH in animal models DFX was most efficacious when administered 2–4 h after ICH at a dose of 10–50 mg/kg depending on species, and this beneficial effect remained for up to 24 h after injury [168]. However, in contrast to studies using the whole-blood model, DFX treatment did not improve outcome in the collagenase model [169]. Therefore, those studies suggest that there are critical differences between these ICH models. Additionally, bipyridine, an iron-chelator, does not lessen intracerebral iron-induced damage or improve outcome after ICH in rats [170]. Perhaps, the current clinical work with iron-chelator will help identify the more clinically predictive model for future neuroprotection studies [171]. Other studies have shown that DFX ameliorates the long-term sequelae of fetal rat matrix hemorrhage [172] and may also reduce hydrocephalus after ICH [173]. Deferoxamine mesylate (DFO), another iron-chelator, improves neurological recovery in animal models of ICH, Moreover, It has been shown that 3 days of 62 mg/kg/day DFO (maximum dose not to exceed 6000 mg/day) is safe and tolerated by intracerebral hemorrhage (ICH) patients [174]. Currently the trial to determine therapeutic benefits of systemic DFO administration in ICH patients is required to form definitive conclusions in further investigation [175].

### 5.4. Synthetic Antioxidant Drugs: Edaravone

Edaravone is a scavenger for oxygen free radicals which has been confirmed to have protective effects in brain injuries in animal experiments. After a rat intraventricular hemorrhage model had been established, immediate treatment with edaravone reduced lipid peroxidation and cerebral edema following intraventricular hemorrhage; continuous treatment for 2 days significantly improved memory impairment and learning [183]. Another set of experiments examined cerebral metabolism around hematoma after cerebral hemorrhage by studying the PET/CT images and found that edaravone treatment significantly improved brain metabolism, alleviated movement disorders, and reduced cerebral edema and apoptosis [148]. Clinical trials with edaravone have also made important progress. Edaravone treatment in patients with aneurysmal SAH reduced late-onset neurological disorders and vascular spasm and improved patient outcomes [178]. In another study of ICH patients, following removal of hematoma with minimally invasive surgery, edaravone treatment significantly improved the NIHSS score and reduced serum MMP-9 levels [184]. However, the results of one large-scale clinical trial suggest that although edaravone treatment improved neurological deficits in some patients, it did not reduce mortality, and long-term clinical benefits were uncertain [185]. Thus, high quality, large-scale clinical trials are still needed to evaluate the benefit of edaravone administration in the treatment of brain bleeding disorders [186] (Table 3).

### 5.5. Targeted Gene Therapy

Gene therapy is a proven effective treatment; however, conventional gene therapy has drawbacks and is limited in practical application. Cheng and colleagues designed a damage-induced vector system which included a hypoxia response element and an antioxidant response element, which could be activated under oxygen deprivation conditions or upon hydrogen peroxide treatment. The advantage of overexpressing Nrf-2 using this vector system to exert neuroprotective effects is that it activates the expression of neuroprotective genes in the nervous system under hypoxia and oxidative stress [187]. Research into a HO-2 (heme oxygenase 2) knockout mouse model with ICH induced by autologous blood injection found that, 4–8 days after ICH, neuron survival in the mice was significantly increased, with a reduction in neuromotor deficits. Therefore, HO-2 is considered a target for oxidative stress treatment for cerebral hemorrhage [188]. However, in a collagenase-induced cerebral hemorrhage model in HO-2 knockout mice, the lesion volume around the hematoma, nerve inflammation, and brain edema were aggravated, with significantly worse neurological defects [189]. This may have been due to the different injury mechanism of the different animal models [190]. In addition to toxicity mediated by iron release, hemin can directly injure cells by oxidative and membrane destabilizing effects [191]. Cultured HO-2 knockout neurons are more vulnerable to inorganic iron, perhaps due to the protective effect of the other products of heme breakdown [192]. The net effect of HO-2 therefore appears to be dependent on the iron binding capacity of the cellular microenvironment. In cerebral hemorrhage the expression time points and location of HO-1 and HO-2 are different [193], and they play different roles for regulating antioxidant gene expression [194]. Heme treatment induces perivascular HO-1 expression and reduces blood brain barrier damage and neurological defects; thus, heme is believed to play a neuroprotective effect through HO-1 [195]. In conclusion, HO plays an important role in SBI after cerebral hemorrhage and regulating HO-1 and HO-2 expression may be a promising therapeutic strategy. Other studies have used neural stem cell transplantation to treat SBI following cerebral hemorrhage, but the host reaction after transplantation led to graft failure and death, which may be related to elevated levels of oxidative stress after transplant. Therefore, Wakai and colleagues transplanted neural stem cells overexpressing SOD1 (copper/zinc-superoxide dismutase) to treat cerebral hemorrhage, which significantly reduced mortality and oxidative stress, speeding up recovery from neurological disorders after ICH [196].

## 6. Conclusions

Multitudinous results have provided information about oxidative stress biomarkers; preclinical and clinical research evidence in ICH further revealed the progress of pathophysiology in cerebral hemorrhage and provided the basis for targeted therapy [5, 93]. However, truly clinically effective treatment strategies are few, mainly because the conflicts of translating preclinical studies into clinical studies have yet to be resolved. The pathological process of survival and rehabilitation in patients after ICH involves complex mechanisms [197]. Secondary brain injuries after ICH include nerve cell toxicity, microglial cell activation, cerebral vascular injury, blood brain barrier damage, arterial venous recanalization and reconstruction, and even other organ damage caused by the interaction of oxidative stress and inflammation [198]. These harmful factors must be effectively addressed to make clinically effective treatment strategies possible.

## Figures and Tables

**Table 1:** Human biomarkers and oxidative stress after ICH.

Biomarkers	Sample	Methods	Value	References
8-iso-Prostaglandin F2*α*	Urinary	Liquid chromatography, tandem mass spectrometry	Independent biomarker of prediction of the risk for incident stroke	[140]

8-iso-Prostaglandin F2*α*	Plasma	Enzyme-linked immunosorbent assay	Disease severity and clinical outcome after acute ICH associated with concentration	[101]

8-OHdG	Plasma	HPLC-electrochemical detector	Level associated with 30-day outcome after ICH	[106]

Bilirubin	Plasma	Reflectance spectrophotometry	Serum bilirubin levels were significantly elevated in the early phases in hemorrhagic stroke	[141]

Vitamin C, uric acid (UA), vitamin E, ubiquinol-10	Plasma	HPLC-electrochemical detector	Lower plasma levels of UA and higher plasma levels of others correlated with the severity of the neurological impairment after ICH	[142]

ROOH	Plasma	HPLC-electrochemical detector	Predictor of poor clinical outcome in sICH survivors	[102]

TAC, TOS	Plasma	Spectrophotometrically	TOS levels increased and TAC levels decreased in acute hemorrhagic stroke	[127]

MDA, myeloperoxidase; erythrocyte glutathione peroxidase; 8-OHdG, leukocyte 8-hydroxy-2′-deoxyguanosine; HPLC, high-performance liquid chromatography; TAC, total antioxidant capacity; TOS, total oxidant status.

**Table 2:** The development for antioxidative treatment of ICH.

Drug name	Administration	Probable mechanism of action or drug targeting	Preclinical or clinical	References
PQQ	Pretreated with 10 mg/kg	Exhibited increased ratio of Bcl-2/Bax, alleviative to activated caspase-3	Rat model of ICH	[129]

Melatonin	150 mg/kg/d, ip; 5 mg/kg/12 h, ip; 15 or 150 mg/kg, ip	Attenuated inflammatory response (IL-1*β*, IL-6, and TNF-*α*, NF-*κ*B, MMP-9, and vEGF); decreased the expression of TLR4 pathway (MyD88, TLR4, NF-*κ*B, and HMGB1); attenuated lipid peroxidation; and activated the Nrf2-ARE pathway	Rat model of SAH or ICH	[112, 143–146]

Sulforaphane	5 mg/kg, ip	Activated Nrf2 in modulating microglia function and hematoma clearance via inducing antioxidative defense components	Rat and mice model of ICH	[50]

Sesamin	30 nmol, icv	Sesamin prevented ICH-induced increase of microglial cells in the perihematomal area, accompanied by the activated p44/42 MAPK pathway	Rat model of ICH	[133]

FeTPPS	30 mg/kg, ip	ONOO^−^ decomposition catalyst; prevented activation of MMP-9 and Hb-induced neurovascular injuries	Rat model of ICH	[31]

Deferoxamine	100 mg/kg, ip	Reduces neuronal death and neurological deficits after ICH in aged rats	Rat model of ICH	[147]

Edaravone	6 mg/kg, SC	Attenuated ICH-induced brain edema, neurologic deficits, and oxidative injury (apurinic/apyrimidinic abasic sites and 8-hydroxyl-2′-deoxyguanosine)	Rat model of ICH	[103]

Edaravone	10 mg/kg, SC	Improved cerebral metabolism around the hematoma by attenuating apoptotic cell death after ICH	Rat model of ICH	[148]

Hydrogen gas	Inhaled, 2.9%	Reduces damage to the blood brain barrier following ICH and improves SBI	Mice model of ICH	[149, 150]

Rosiglitazone	0.5 mg, infused into the hematoma regions	A remarkable decrease in perihematomal levels of PPAR*γ*, MMP-9, BBB permeability, and BWC following minimally invasive surgery for ICH treatment	Rabbits model of ICH	[151]

Minocycline	45 mg/kg, ip	Reduces iron accumulation and inhibits microglia activation contributing to brain damage after ICH via suppressing MMP-9	Rat model of ICH	[152, 153]

Atorvastatin	2, 5, and 10 mg/kg, orally	Decreasing the brain injury and protecting neurons in ICH involving suppression of TNF-*α*/MMP-9 and upregulation of IL-10	Rat model of ICH	[154, 155]

PQQ, pyrroloquinoline quinine; MMP-9, matrix metallopeptidase 9; TLR4, Toll-like receptor 4; iNOS, inducible nitric oxide synthase; MyD88, myeloid differentiation factor 88; ip, intraperitoneal injection; icv, intracerebroventricular injection; SC, subcutaneous injection.

**Table 3:** Potential medications that target oxidative stress in patients after hemorrhagic stroke.

Drug name	Clinical trials	Different types of intracerebral hemorrhage	Action mechanism	Outcome	References
NXY-059	NCT00075959 (CHANT)	ICH	Free radical-trapping agent	No benefit	[176]

Deferoxamine mesylate	NCT01662895 (Hi-Def)	ICH	Iron-chelator	Phase II clinical trial	[177]

Hydrogen-rich fluid	UMIN000014696	SAH	Delayed cerebral ischemia and cerebral vasospasm	Ongoing	[160]

Edaravone	—	SAH	Free radical scavenger	Preliminary	[178]

Simvastatin	NCT01077206 ISRCTN75948817	SAH	Lipid-lowering therapy	No benefit	[179, 180]

Lipid-lowering medication	NCT00226096 and NCT00716079 (INTERACT)	ICH	Lipid-lowering therapy	No benefit	[181]

Statin	NCT00221104	SAH	Lipid-lowering therapy	No benefit	[182]

ICH, intracranial hemorrhage; SAH, subarachnoid hemorrhage.
